# The first complete mitochondrial genome of the micro-whip-scorpion *Schizomus zhensis* (Arachnida: Schizomida) and phylogenetic analysis

**DOI:** 10.1080/23802359.2022.2067499

**Published:** 2022-04-25

**Authors:** Yun Bu, Yan Gao, Nerivania Nunes Godeiro

**Affiliations:** Shanghai Natural History Museum, Shanghai Science & Technology Museum, Shanghai, China

**Keywords:** Mitogenome, schizomids, arachnids, phylogeny

## Abstract

The first complete mitochondrial genome of the micro-whip-scorpion (*Schizomus zhensis*) was assembled from Illumina-based whole genome sequencing. The circular genome is 14,309 bp in length with 13 protein coding genes, 22 tRNA, 2 rRNAs, and a control region. The A + T content of the mitogenome is 76.6%. Maximum likelihood phylogenetic analyses placed *Schizomus zhensis* as a sister group of (Thelyphonida + Amblypygi), but more mitogenomes of Tetrapulmonata need to be included in further analyses for a conclusive decision about the group systematics.

## Introduction

Arachnids are a fairly diverse arthropod group in the world with about 80,000 species described up to know (https://www.catalogueoflife.org/). However, genomic studies of arachnids are still insufficient. There are only 181 mitogenomes of arachnids species released in GenBank (accessed November 2021) and most of them are ticks and mites, data for the order Schizomida were still missing. Schizomids or micro-whip-scorpions belong to a small order of Arachnida with 361 species known in the world (Monjaraz-Ruedas et al. 2020). In order to fill in the gap of genomic studies in this special group, we sequenced the first complete mitochondrial genome of a micro-whip-scorpion and analyzed its phylogenetic position.

Specimens of *Schizomus zhensis* Chen and Song 1996 were collected from Daji Mountain (31.5300 N, 120.2032 E), Wuxi City, Jiangsu Province of East China by Yun BU on 8 July 2021. Specimens were deposited at Shanghai Natural History Museum (Yun BU, email: buy@sstm.org.cn.) under the voucher number SC-JS-2021001.One individual was used for DNA extraction and whole-genome amplification. All laboratory experiments including library construction and sequencing were performed by Shanghai Yaoen Biotechnology Co., Ltd., China. Illumina NovaSeq platform was used for sequencing paired-end reads with 150 bp length, producing approximately 10 G of data. The mitogenome was assembled de novo using NovoPlasty v3.8.3 (Dierckxsens et al. 2020) with kmer value 25 and a COI partial sequence from a species belonging to the same family (*Stenochrus sbordonii* Brignoli) was used as a seed (accession number KY017755). The identity and position of the 13 PCGs, 22 tRNA, and 2 rRNA genes were determined using MitoZ v2.4-alpha (Meng et al. 2019) and MITOS web server (Bernt et al. 2013). The finished mitogenome was deposited in NCBI with the accession number OL544939.

**Figure 1.**
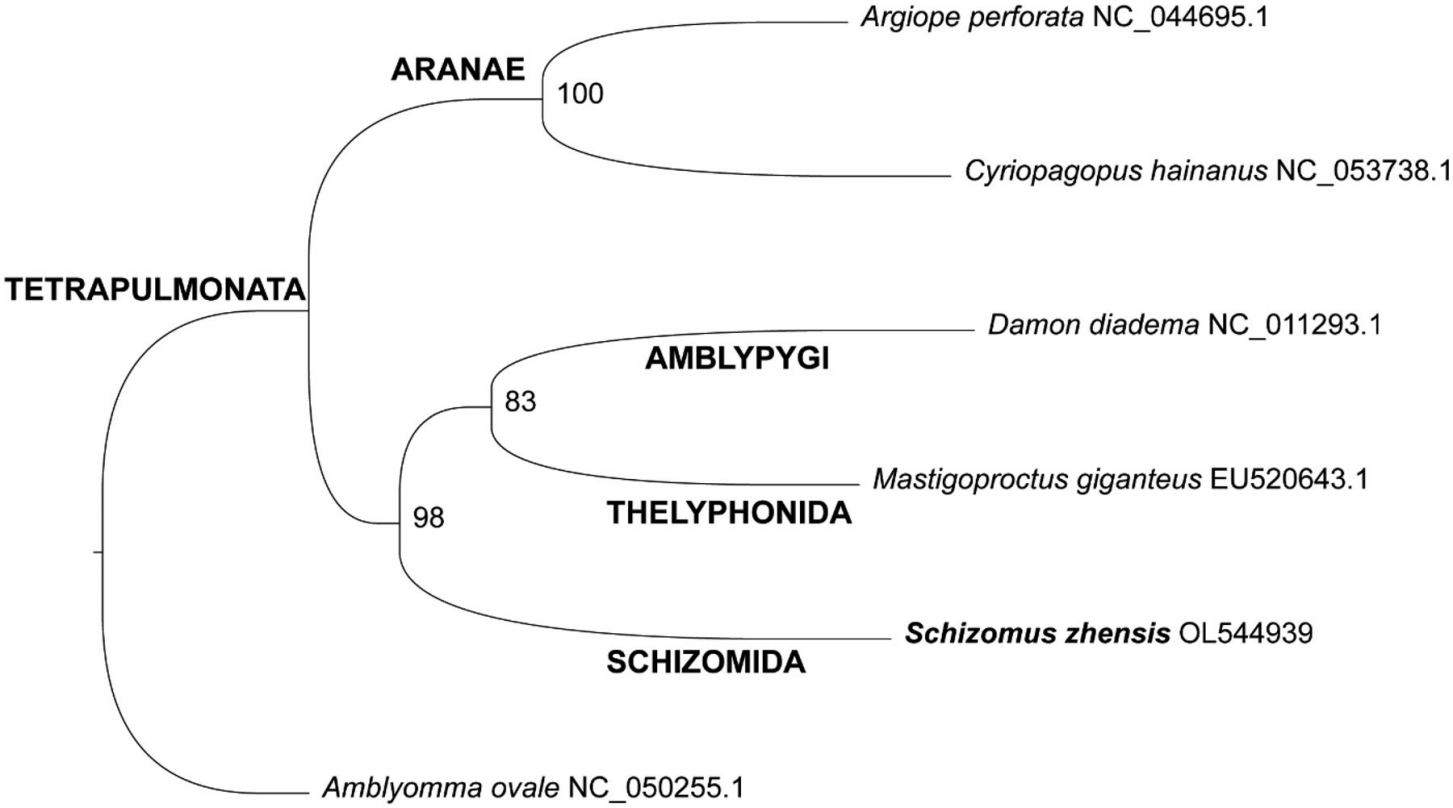
Phylogenetic placement of *Schizomus zhensis* based on maximum likelihood inference. Ultrafast bootstrap support is indicated in each node and GenBank accession numbers are presented in the branches.

Previously to the phylogenetic analyses, mitogenomes sequences of four taxa of Tetrapulmonata and one Acari (*Amblyomma ovale* Sars) were downloaded from GenBank. All accession numbers are listed in Figure 1. The newly assembled mitogenome of *Schizomus zhensis* (14,309 bp) was included and the final dataset comprised six species. Nucleotide sequences of all 13 protein coding genes were aligned using MAGUS (Smirnov and Warnow 2020) and BMGE v1.12 (Criscuolo and Gribaldo 2010) was used to trim the alignments. FASconCAT-G v1.04 (Kück and Longo 2014) was used to concatenate the sequences and a phylogenetic matrix with a partition scheme was created. The final matrix comprised six taxa with four partitions and 7,227 sites. Bayesian phylogenetic inference was performed using PhyloBayes MPI Version 1.5a (Lartillot et al. 2013), with CAT-GTR model, two chains were run until the likelihood had satisfactorily converged (maxdiff < 0.1). Maximum Likelihood inference was performed using IQ-Tree v2.0.7 (Minh et al. 2020), ultrafast bootstrap 1,000 replicates (Hoang et al. 2018), and SH-aLRT support. Model Finder (Subha Kalyaanamoorthy et al. 2017) selected the best partition scheme and GTR + F substitution model for all partitions. The phylogenetic tree was visualized and edited in FigTree v1.4.2 (available on https://tree.bio.ed.ac.uk/software/figtree/). The resulting topology based on ML (Figure 1) suggested the position of Schizomida as a sister group of (Amblypygi + Thelyphonida) with high bootstrap support (98%). Bayesian analyses placed the new mitogenome as a sister group of Amblypygi with a bit lower support (0.95). The lack of complete mitogenomes belonging to Tetrapulmonata made our result inconclusive, considering that Schizomida is currently recognized as sister group of Thelyphonida (Ballesteros et al. 2019).

## Data Availability

The mitogenome sequence data that support the findings of this study are openly available in GenBank of NCBI at (https://www.ncbi.nlm.nih.gov/) under the accession number OL544939. The associated **BioProject**, **SRA**, and **Bio-Sample** numbers are PRJNA783377, SRR17035428, and SAMN23429391, respectively.
